# Variations of dry eye disease prevalence by age, sex and geographic characteristics in China: a systematic review and meta-analysis

**DOI:** 10.7189/jogh.08.020503

**Published:** 2018-12

**Authors:** Peige Song, Wei Xia, Manli Wang, Xinlei Chang, Jingpin Wang, Shuai Jin, Jiawen Wang, Wei Wei, Igor Rudan

**Affiliations:** 1 Centre for Global Health Research, Usher Institute of Population Health Sciences and Informatics, University of Edinburgh, Edinburgh, Scotland, United Kingdom; 2 School of Nursing, University of Hong Kong, Hong Kong, China; 3 School of Public Health, Peking University, Beijing, China; 4 School of Nursing, Peking University, Beijing, China; 5 Institute of Medical Humanities, Peking University, Beijing, China

## Abstract

**Background:**

Dry eye disease (DED) is one of the most prevalent ocular diseases in the world. In China, new lifestyles driven by information technology and the rapid ageing process have brought DED a severe public health concern. The aim of our study was to obtain the pooled prevalence of DED in China and explore its potential correlates.

**Methods:**

A comprehensive systematic review was conducted to identify all relevant literature published since 1990. Meta-analysis and meta-regression approaches were adopted to estimate the prevalence of DED. The number of people with DED was obtained by multiplying the corresponding demographic data in 2010.

**Results:**

Advanced age, female sex and larger latitude were significant risk factors for DED by symptoms and signs, whereas only advanced age was positively associated with an increased prevalence of DED by symptoms. In 2010, the prevalence of DED by symptoms and signs were 13.55% (95% CI = 10.00-18.05) and that of DED by symptoms was 31.40% (95% CI = 23.02-41.13) in Chinese people aged 5-89 years, corresponding to a total of 170.09 million (95% CI = 125.52-226.63) and 394.13 million (95% CI = 288.99-516.30) affected individuals respectively.

**Conclusions:**

The huge burden of DED in China calls for more public health attention and actions. Improved epidemiological studies on DED prevalence are still urgently needed.

Dry eye disease (DED), a multifactorial tear deficiency disorder of the ocular surface, is one of the most prevalent ocular diseases in the world [1-3]. In daily ophthalmological practice, DED is a frequently encountered ocular problem, it is characterised by different combinations of excessive tear evaporation, decreased tear production and poor tear quality [3,4]. Affected individuals are generally plagued by various symptoms, such as photophobia, fatigue, itchiness, burning, irritation and visual disturbance [1,5]. Although these debilitating symptoms do not lead to severe visual impairment and blindness, they are highly related to the reduction in vision-related quality of life and interfere with daily activities [6-8]. Furthermore, the limited ability to produce tears may result in an increased risk of ocular infection and ocular surface damage, or even abrasion or corneal ulceration in severe cases [5,9].

There is still no gold diagnostic procedure of DED [9,10], and the treatment of DED is only a temporarily symptomatic relief and improvement [3,9]. Common symptom-based questionnaires include the Ocular Surface Disease Index and the Dry Eye Questionnaire [1]. In addition to symptoms, signs are also essential components in virtually all the definitions of DED, common tests of DED include the Schirmer’s I test (SIt), tear film breakup time (TBUT), fluorescein staining (FS) of the cornea and conjunctiva [11]. Previous epidemiological evidence has revealed an overall DED prevalence of 5% to as high as 50% in general population across the world, the large variation may come from the disparities of diagnostic criteria, as well as characteristics of the investigated population and etiological factors [4,12-17]. Important risk factors for DED were also identified, including advanced age, female sex, use of exogenous oestrogen and visual display terminals (VDTs) [1,12,13,18-20]. Other environmental factors, such as low relative humidity, have also been suggested as probable contributors of DED but still need further validation [1,21].

According to the 2017 report of the Tear Film and Ocular Surface Society (TFOS) Dry Eye Workshop (DEWS) Epidemiology subcommittee, Asian race was suggested as a significant risk factor for DED [17]. For China, the largest developing country, new lifestyles driven by information technology and the rapid ageing process have brought DED a severe public health concern in both young and old generations [4,22,23]. The latest systematic review and meta-analysis of DED prevalence in China conducted by N Liu, et al. revealed a pooled DED prevalence of 17.0%, by using prevalence estimates from 12 individual studies. However, two out of the 12 studies reported the prevalence of symptomatic DED, whereas 10 reported the prevalence of DED diagnosed by both symptoms and signs [23]. Given the fact that studies based on symptom-diagnosis report higher prevalence rates of DED than those involved both symptoms and signs [15], it is reasonable to believe that their estimate of DED prevalence is problematic. Furthermore, the relation between patient-reported symptoms and clinical signs has been proven to be poor and inconsistent [24-26], it is, therefore, necessary to synthesise the DED prevalence in different diagnostic groups separately. In view of the geo-epidemiology of DED, the large geographical area of China provides a very good opportunity to explore the geographic variations of DED prevalence within the same country. In this study, we conducted a systematic review of the prevalence of DED, with a focus on the difference in diagnostic methods. To better understand the probable risk factors, we investigated the differences in prevalence by age, sex, setting and geographic factors.

## METHODS

### Search strategy and selection criteria

This study is in accordance with the Preferred Reporting Items for Systematic reviews and Meta-Analysis (PRISMA) guidelines [27]. No protocol for this systematic review was pre-registered. A comprehensive systematic review was conducted to identify all relevant literature published from January 01, 1990 to November 27, 2016. First, three Chinese and three English bibliographic databases, namely, Wanfang, China National Knowledge Infrastructure (CNKI), Chinese Biomedicine Literature Database (CBM-SinoMed), Medline, PubMed and Embase, were searched, the search terms included “prevalence” or “incidence” or “epidemiology” or “morbidity” or “mortality”, combined with “dry eye” and “China or Chinese”, in the forms of free words or controlled vocabulary (ie, medical subject headings). Different search languages and strategies were developed for different databases to adapt to their specific characteristics (Table S1 in **Online Supplementary Document(Online Supplementary Document)**). No language restrictions were applied. Second, snowball searching was performed to further identify potentially relevant studies from the reference lists of articles in the first step.

All citations were independently reviewed by two researchers (MW and XC) in two stages: screening titles and abstracts followed by an appraisal of full-text articles. Criteria for inclusion were epidemiological investigations of DED in general Chinese population. Studies that were conducted in subpopulations, such as people with specific diseases (eg, diabetes, hypertension, rheumatoid arthritis, lupus, Sjögren’s syndrome, thyroid associated diseases), were excluded because of their poor representativeness of general population. All included studies should have a clear assessment and diagnostic method of DED. For multiple publications of the same study, the one with the most details was kept for further analysis.

### Data extraction and quality assessment

The same two researchers (MW and XC) independently extracted the following information from the included studies: study characteristics (eg, author[s], publication year, study design, assessment and diagnostic method, study site, study year), population characteristics (eg, age, sex) and the outcome measures (the number of participants and DED cases). For studies that reported prevalence in school children, we unified the age range of middle school students as 13-15 years, and high school students as 16-18 years according to the national education schemes [28]. The geographic information (latitude and longitude) of the investigation sites in all included studies was obtained by using the Google Maps GPS coordinates (http://www.gps-coordinates.net/).

In this study, DED was categorised into two diagnostic groups: “DED by symptoms and signs” and “DED by symptoms”, according to the diagnostic methods reported in individual studies. DED by symptoms and signs was defined as a positive symptom with at least one positive clinical sign tested by BUTS≤10s (5s) or SIt ≤10s (5s) or FS≥1 (2 or 3). Whereas DED by symptoms only replied on the presence of self-reported positive symptoms. If more than one outcome measure were reported in different sub-groups (eg, different age or sex groups) within the same study, data were extracted for each subgroup separately. The quality of included studies was assessed according to the Reporting of Observational Studies in Epidemiology (STROBE) guideline [29]. Sample population, sample size, participation rate, outcome assessment, and analytical methods were assessed by scoring, with 0 for high risk and unclear, 1 for moderate risk and 2 for low risk. The total score of these five core components represented the overall bias risk of each study (Table S2 in **Online Supplementary Document(Online Supplementary Document)**) [30,31]. Discrepancies at each step of both screening and data extraction were discussed and resolved with the help of a third independent researcher (PS) until consensus was reached.

### Statistical analysis

Prevalence of DED in each included study was calculated from the raw proportion and stabilised by using the logit transformation [32]. The heterogeneity between studies was tested by Cochran's Q statistic (Q-test) and quantified by *I*^2^ index. A *P* < 0.05 in the Q-test indicates significant heterogeneity, and *I*^2^ index represents the percentage of between-study variance due to heterogeneity rather than chance, where values of 25% or less correspond to low heterogeneity, near 50% indicate moderate heterogeneity and near 75% or larger reflect high heterogeneity [33,34]. In the case of significant heterogeneity (*P* < 0.05 or *I*^2^>50%), a random-effects meta-analysis (DerSimonian and Laird method) was adopted to generate the pooled prevalence and 95% confidence intervals (CIs), otherwise, a Mantel-Haenszel fixed-effects model was adopted [35]. Leave-one-out sensitivity analysis was conducted to assess the robustness of the pooled results. Funnel plots were drawn to address publication bias visually, Begg’s rank correlation test and Egger’s linear regression were conducted to quantify the degree of publication bias [36,37].

The source of heterogeneity was explored by meta-regression. First, we did univariable meta-regression to test the individual association of prevalence estimates and relevant factors, which included age, sex (male vs female), setting (urban vs rural), study year and geographic indicators (latitude and longitude). Factors that were significantly associated with the prevalence of DED in the univariable meta-regression were subsequently included in the multivariable meta-regression analysis. To visually inspect the goodness of the final multivariable regression, diagnostic plots were generated, including predicted vs standardised residuals, Quantile-Quantile (QQ) plot and histogram of residuals.

Finally, based on the multivariable regression model, age and sex-specific prevalence of DED was calculated for the whole country and six geographic regions (East China, North China, Northeast China, Northwest China, South Central China, Southwest China) (Figure S1 in **Online Supplementary Document(Online Supplementary Document)**) respectively. The number of people with DED in 2010 was calculated by multiplying the age and sex-specific DED prevalence with the corresponding population data obtained from the 6th national census [38]. All analyses were performed using R, version 3.3.0 (R Foundation for Statistical Computing, Vienna, Austria), the China map was obtained from the Global Administrative Areas database (GADM, 2015, version 2.0; www.gadm.org) and all maps were drawn by ArcMap version 10.1 (Environmental Systems Research Institute, Redlands, CA). A *P* < 0.05 indicated statistical significance and all *P*-values were 2-sided.

## RESULTS

### Search and selection results

**Figure 1:**
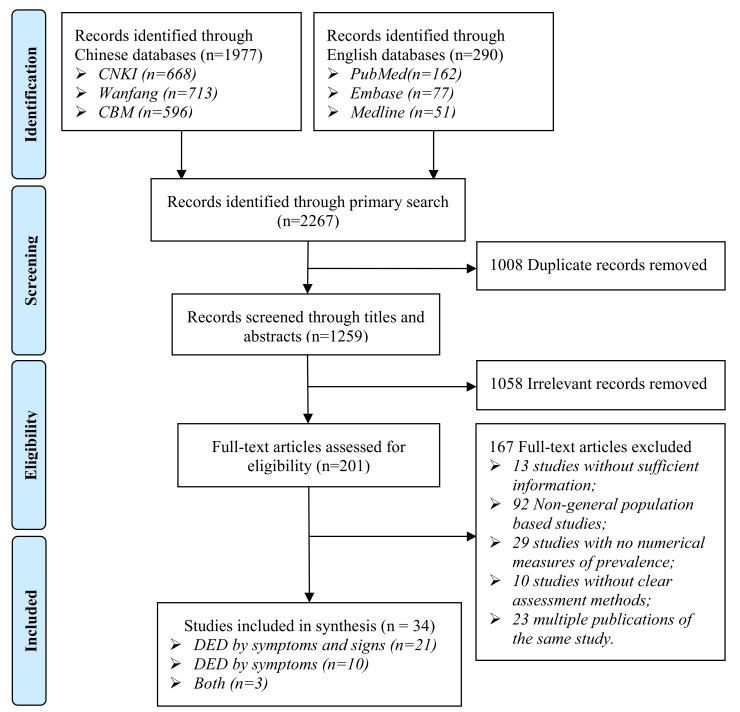
PRISMA of the study selection process.

A total of 2267 citations were identified by the primary search. After removing 1008 duplicates, the titles and abstracts of 1259 studies were screened for relevance. Then the full texts of 201 studies were assessed for eligibility, finally, 34 studies met the inclusion criteria, of which 21 reported the prevalence of DED by symptoms and signs, ten reported the prevalence of DED by symptoms and three reported both the prevalence of DED by symptoms and signs and that of DED by symptoms (**Figure 1**).

### Study characteristics and quality assessment

A summary of the main characteristics of the 34 included studies is listed in **Table 1**, and the detailed information can be found in Table S3 in **Online Supplementary Document(Online Supplementary Document)**. 24 studies provided prevalence estimates of DED by symptoms and signs, where a total of 10113 individuals were identified as DED patients by symptoms and signs out of 65959 participants. All the 24 studies were published in this century, with the majority being published in the last seven years (2010-2016) (n = 19, 79.17%), half being conducted in urban areas (n = 12, 50%) and most providing prevalence for both males and females (n = 23, 95.83%).

**Table 1:** Summary of the main characteristics of the included studies

Characteristics of study	Studies with prevalence of DED by symptoms and signs (n = 24, %)	Studies with prevalence of DED by symptoms (n = 13, %)
**Year published:**		
2000-2009	5 (20.83)	7 (53.85)
2010-2016	19 (79.17)	6 (46.15)
**Setting**:		
Urban	12 (50.00)	2 (15.38)
Rural	2 (8.33)	2 (15.38)
Mixed	10 (41.67)	9 (69.23)
**Sex:**		
Both	23 (95.83)	11 (84.62)
Mixed	1 (4.17)	2 (15.38)
**Sample** size:		
300-1000	10 (41.67)	2 (15.38)
1001-2000	3 (12.50)	9 (69.23)
2001-5000	8 (33.33)	1 (7.69)
>5000	3 (12.50)	1 (7.69)
**Quality score:**		
10	9 (37.50)	2 (15.38)
9	7 (29.17)	6 (46.15)
8	0 (0.00)	0 (0.00)
7	2 (8.33)	4 (30.77)
6	6 (25.00)	1 (7.69)

DED – dry eye disease

In the 13 studies that provided prevalence estimates of DED by symptoms, a total of 28277 participants were included, among whom 8955 were with DED symptoms. More than half of the studies were published during 2000 to 2010 (n = 7, 53.85%), mainly from mixed areas (rural and urban together) (n = 9, 69.23%), and reported prevalence for both males and females (n = 11, 84.62%).

All studies were rated with assessment scores of at least 6. For studies with prevalence estimates of DED by symptoms and signs and those by symptoms, more than half received scores of 10 or 9. The detailed assessment of study quality can be found in Table S4 in **Online Supplementary Document(Online Supplementary Document)**.

### Prevalence of DED by symptoms and signs

#### Meta-analysis and meta-regression

Meta-analysis of the 24 studies reporting the prevalence of DED by symptoms and signs revealed significantly high heterogeneity between studies (*I*^2^ = 99.3%, *P* < 0.001). Therefore random-effects model was adopted, as shown in Figure S2 in **Online Supplementary Document(Online Supplementary Document)**, the pooled prevalence of DED by symptoms and signs was 18.48% (95% CI = 14.76-22.88) from the included 24 studies. According to the leave-one-out sensitivity analysis (Figure S3 in **Online Supplementary Document(Online Supplementary Document)**), the pooled prevalence of DED by symptoms and signs varied from 17.76% (95% CI = 14.14-22.06) to 19.30% (95% CI = 14.44-23.85), no single study significantly influenced the liability and stability of the overall pooled prevalence in the meta-analysis. Potential publication bias was examined by funnel plot, Egger’s test and Begg’s test respectively (Figure S4 in **Online Supplementary Document(Online Supplementary Document)**), although the asymmetrical shape of funnel plot suggested potential publication bias, Egger’s test (*t* = 0.688, *P* = 0.498) and Begg’s test (z = -0.496, *P* = 0.620) didn’t reveal a risk of publication bias.

In the univariable meta-regression analyses (**Table 2**), setting (urban vs rural), study year and longitude were not significantly associated with the prevalence of DED by symptoms and signs, whereas age, sex and latitude were all statistically significant covariates. All significant covariates were then included in the subsequent multivariable meta-regression. Diagnostic plots for this meta-regression model are demonstrated in Figure S5 in **Online Supplementary Document(Online Supplementary Document)**.

**Table 2:** Univariable and multivariable meta-regression models of covariates related to the prevalence of DED by symptoms and signs*

Moderator	Number of studies	Number of data points	β	95% CI	*P*-value
**Univariable meta-regression:**					
Intercept	24	112	-1.409	-1.656 to -1.162]	<0.001
Age	24	112	0.020	0.018 to 0.022	<0.001
Sex-Male	23	98	-0.252	-0.297 to -0.207]	<0.001
Setting-Rural	14	68	0.003	-1.065 to -1.071	0.996
Study year	24	112	0.031	-0.063 to -0.126	0.518
Latitude	24	112	0.056	0.019 to 0.094	0.003
Longitude	24	112	-0.001	-0.023 to 0.022	0.965
**Multivariable meta-regression:**					
Intercept	23	98	-4.708	-6.200 to -3.217]	0.015
Age	23	98	0.020	0.018 to 0.022	<0.001
Sex-Male	23	98	-0.240	-0.285 to -0.195]	<0.001
Latitude	23	98	0.073	0.029 to 0.117	0.001

CI – confidence interval

*Coefficients represent log odds ratios (ORs).

#### Estimates of prevalence and cases

The age- and sex-specific prevalence of DED by symptoms and signs was estimated for China and the six Chinese regions (Table S5 in **Online Supplementary Document(Online Supplementary Document)**). In all the six regions and the whole country, the prevalence of DED by symptoms and signs was consistently higher in females than in males, and increased steadily with age. By multiplying the corresponding population in 2010, the overall prevalence of and the number of people with DED by symptoms and signs in people aged 5-89 years are listed in **Table 3**. In 2010, the overall prevalence of DED by symptoms and signs was 13.55% (95% CI = 10.00-18.05) in China, and ranged from 8.91% (95% CI = 6.22-12.60) in South Central China to 26.42% (95% CI = 17.79-37.25) in Northeast China, which demonstrated a positive trend with increased latitudes. The number of people aged 5-89 years that had DED by symptoms and signs was 170.09 million (95% CI = 125.52-226.63), and the lowest in Northwest China (16.24 million, 95% CI = 12.50-20.78) and highest in East China (45.52 million, 95% CI = 35.77-57.44).

**Table 3:** Sex-specific prevalence and the number of people with DED by symptoms and signs in China and the six Chinese regions in 2010

Region	Prevalence of DED by symptoms and signs (%, 95% CI)			People with DED by symptoms and signs (million, 95% CI)		
**Male**	**Female**	**Overall**	**Male**	**Female**	**Overall**
North China	17.81	21.73	19.72	14.23	16.50	30.73
	(13.24-23.51)	(16.45-28.11)	(14.80-25.75)	(10.57-18.78)	(12.49-21.34)	(23.06-40.12)
Northeast China	24.06	28.82	26.42	12.83	15.01	27.84
	(15.98-34.48)	(19.65-40.08)	(17.79-37.25)	(8.52-18.39)	(10.23-20.87)	(18.75-39.25)
East China	10.93	13.68	12.29	20.55	24.97	45.52
	(8.52-13.91)	(10.82-17.14)	(9.65-15.50)	(16.01-26.15)	(19.75-31.29)	(35.77-57.44)
South Central China	7.89	9.99	8.91	14.21	17.10	31.32
	(5.46-11.25)	(7.01-14.03)	(6.22-12.60)	(9.84-20.27)	(12.00-24.02)	(21.84-44.29)
Southwest China	9.06	11.34	10.18	8.39	10.06	18.45
	(6.63-12.26)	(8.41-15.11)	(7.50-13.65)	(6.13-11.34)	(7.46-13.40)	(13.59-24.75)
Northwest China	16.12	19.66	17.84	7.54	8.70	16.24
	(12.30-20.82)	(15.25-24.95)	(13.74-22.83)	(5.75-9.74)	(6.75-11.04)	(12.50-20.78)
China	12.14	15.02	13.55	77.75	92.34	170.09
	(8.87-16.34)	(11.17-19.84)	(10.00-18.05)	(56.84-104.67)	(68.68-121.96)	(125.52-226.63)

### Prevalence of DED by symptoms

#### Meta-analysis and meta-regression

As shown in Figure S6 in **Online Supplementary Document(Online Supplementary Document)**, significant high heterogeneity existed between the 13 studies that provided prevalence of DED by symptoms (*I*^2^ = 99.7%, *P* < 0.001). The pooled prevalence of DED by symptoms was 38.89% (95% CI = 27.06-52.20) using the random-effects model. When removing one single study at one time, the pooled prevalence of DED by symptoms ranged from 35.80% (95% CI = 25.65-47.40) to 41.70% (95% CI = 31.53-52.63), the liability and stability were not significantly influenced (Figure S7 in **Online Supplementary Document(Online Supplementary Document)**). Visually inspection of the funnel plot suggested potential publication bias, but neither Egger’s test (*t* = 1.831, *P* = 0.094) nor Begg’s test (z = 0, *P* = 1.0) significantly indicated any publication bias (Figure S8 in **Online Supplementary Document(Online Supplementary Document)**).

Only age was revealed as a significant covariate with the prevalence of DED by symptoms in the univariable meta-regression analyses (**Table 4**). The final meta-regression was constructed by taking the variation of age, and the diagnostic plots are shown in Figure S9 in **Online Supplementary Document(Online Supplementary Document)**.

**Table 4:** Univariable and multivariable meta-regression models of covariates related to the prevalence of DED by symptoms*

Moderator	Number of studies	Number of data points	β	95% CI	*P*-value
**Univariable meta-regression:**					
Intercept	13	53	-0.554	-0.987 to -0.122	0.012
Age	13	53	0.026	0.024 to 0.029	<0.001
Sex-Male	11	42	-0.658	-1.836 to 0.521	0.274
Setting-Rural	–	–	–	–	–
Study year	13	53	0.000	-0.112 to 0.113	0.997
Latitude	13	53	0.064	-0.016 to 0.143	0.116
Longitude	13	53	-0.021	-0.070 to 0.029	0.421
**Multivariable meta-regression:**					
Intercept	13	53	-1.817	-2.277 to -1.356]	<0.001
Age	13	53	0.026	0.024-0.029	<0.001

CI – confidence interval

*When insufficient data were available for the comparison between urban and rural settings, it is indicated by “–“.

#### Estimates of prevalence and cases

The age-specific prevalence of DED by symptoms is listed in Table S6 in **Online Supplementary Document(Online Supplementary Document)**, by multiplying the Chinese population data, the overall prevalence of DED by symptoms was 31.40% (95% CI = 23.02-41.13) in people aged 5-89 years, and the corresponding number of people with DED by symptoms was estimated as 394.13 million (95% CI = 288.99-516.30).

## DISCUSSION

In this study, we presented a comprehensive estimate of DED prevalence in general Chinese population in 2010 by systematic reviewing all published epidemiological information on DED, and explored its variations in various groups with different demographic and geographic factors. To date, the variation of definitions and parameters in the existing studies has restricted the synthesis of DED prevalence to a large extent, although DED has been widely recognised as a common ocular disorder [16,19,31,39,40]. Our study is the second study to generate an overall estimate of DED prevalence in general Chinese population by using meta-analysis approach, but it is the first effort to report the prevalence of DED by symptoms and signs and DED by symptoms in China separately, which is of both clinical and epidemiological importance.

The merits of our study mainly arise from the comprehensive systematic review approach, which resulted in a more than doubled amount of studies in our final analysis than in the study conducted by N Liu, et al. [23]. In addition, multiple data points from the same study provided much more information than taking only one estimate from each study, our ability to generate estimates of DED prevalence in a broad age range was guaranteed. Furthermore, although significant heterogeneity existed between the included studies, this high variation may be largely explained by the uneven distribution of various risk factors in investigated sites, such as older age, female sex and low relative humidity [1,12,13,18-21]. Our estimates of DED prevalence were made by taking the significantly associated factors into account, rather than simply being pooled together, this measure also largely improved the reliability of our national and regional estimates [41,42].

In the current study, we revealed an overall prevalence of DED by symptoms and signs of 13.55% and a much higher prevalence of DED by symptoms of 31.40%. Compared with the study conducted by N Liu, et al., which reported a pooled DED prevalence of 17.0% by mixing both DED by symptoms and signs and DED by symptoms together, it is reasonable to observe a lower prevalence of DED by symptoms and signs and a higher prevalence of DED by symptoms in our study [23]. The higher prevalence of DED by symptoms generated in this study (31.40% in people aged 5-89 years) than those reported in the United States of American (15.2% in adults aged 21 years and above) and England (20.8% in females aged 20 years and above) confirms the notion that Asians were at a higher risk of DED than Caucasians, as suggested by the TFOS DEWS II Epidemiology Report 2017 [17].

Our study revealed that advanced age was a significant risk factor for both DED by symptoms and signs and DED by symptoms, which confirmed the assumption that DED was an age-related disorder, this finding is in line with previous evidence [12,23,43]. The reduction of tear secretion and an increase of evaporation with biological ageing may be the core explanation [3,43]. For the prevalence of DED by symptoms and signs, female sex was also found to be a positive covariate. The persisted sex difference across all ages has also been reported in some previous studies, which might be a combined result of different hormonal effects on the ocular surface, lacrimal gland, etc., however, this variation between sexes has not become a universally acknowledged notion in previous studies [12,15,44,45], this may be caused by different definitions of DED in their investigations, in our study, no sex difference was observed for the prevalence of DED by symptoms. More studies are still needed to explore if DED is a more prevalent condition among females, with a focus on different types of DED. In the exploration of geo-epidemiology of DED prevalence, increased latitude was found to be a risk factor for DED by symptoms and signs in our study, given that low relative humidity is a well-proven risk factor for DED [3,46], the negative relation of relative humidity and latitude in China may be the reason for this spatial distribution of DED by symptoms and signs [23,47,48]. The absence of a geographic variation of DED by symptoms may be due to the limited number of unique study sites, which was only 11. With more epidemiological studies on DED emerging, the geo-epidemiology study of DED by symptoms can become possible in the foreseen future.

The large magnitude of DED cases represents a huge public health burden in China, in this study, it was estimated that 170.09 million individuals were living with DED by symptoms and signs and 394.13 million with DED by symptoms. This figure indicates that the number of Chinese people experiencing symptoms of DED in 2010 was even larger than the contemporary overall population in the United States. In this modern society where prolonged visual tasking plays an important role in daily activities, the visual well-being is especially essential [6,49]. The uncomfortable symptoms together with diminished visual performance and quality of life, therefore, represent a huge burden on the whole society and health care system [1,50]. With the rapid ageing trend in China, this burden of this age-related condition will predictably be even larger in the coming years.

Our study also has several potential limitations. First, although a comprehensive systematic review was conducted to retrieve all potential studies, all of our included studies were published, the absence of unpublished studies may bring bias in our analyses and final estimates. Second, despite our best efforts to minimise variations by applying strict criteria, significant large heterogeneity was still observed among the included studies. In meta-regression, only a limited assortment of group-level variables was examined, namely, age, sex (male vs female), setting (urban vs rural), study year and geographic indicators (latitude and longitude), according to previous studies, the prevalence of DED is especially high in people with Sjögren's syndrome or visual display terminal users, women at perimenopause and postmenopause may also be at higher risk of developing DED [6,12,19]. However, because of the lack of availabile information, we could not explore the impact of individual-level variables in our meta-regression approaches. Third, the included studies were not from across the whole country, which limited our ability to generate estimates of DED prevalence in every single province, therefore we only portrayed an overall picture of the DED burden for the whole country, and the six geographic regions at best, regardless of the fact that variations may exist within the country or a region. Fourth, latitude was assessed as a significant covariate of DED prevalence, to estimate the prevalence and burden of DED in different geographic regions, DED cases were calculated based on the Chinese demographic data in 2010, which provided the latest demographic data stratified by geographic areas. This, however, represents a significant time-lag for assessing the latest prevalence and burden of DED in China in 2018.

## CONCLUSIONS

To conclude, our study reveals that DED is a prevalent disorder in the general Chinese population, and its impact and importance will grow with the demographic ageing trend. In 2010, the prevalence of DED by symptoms and signs were 13.55% and that of DED by symptoms was 31.40% in Chinese people aged 5-89 years, corresponding to a total of 170.09 million and 394.13 million affected individuals respectively. Advanced age, female sex and larger latitude were significant risk factors for DED by symptoms and signs, whereas only advanced age was positively associated with an increased prevalence of DED by symptoms. The huge burden of DED in China calls for more public health attention and actions. Improved epidemiological studies on DED prevalence are still needed.
