# Supplementary material for: Variations of dry eye disease prevalence by age, sex and geographic characteristics in China: a systematic review and meta-analysis
Source: J Glob Health. 2018 Sep 3;8(2):020503. doi: 10.7189/jogh.08.020503 (PMC6122008; doi:10.7189/jogh.08.020503)
Supplement: Online Supplementary Document [file jogh-08-020503-s001.pdf]

Online Supplementary Document

Song et al. Variations of dry eye disease prevalence by age, sex and geographic characteristics in China: a systematic review and meta-analysis

J Glob Health 2018;8:020503

Table S1 Search strategies in six bibliographic databases.

Database	Access date	Subject category	Sub-database	Search terms	Publication date	Search method
**CNKI**	27/11/2016	Medicine & Public Health	Journal, Featured journal, Doctoral dissertation, Master dissertation, Domestic conferences, International conferences	(SU % '干眼' + '干眼症' + '干眼病') AND (SU % '发病率' + '发生率' + '患病率'+ '罹患率' + '现患率'+ '死亡率' + '病死率'+ '流行' + '负担'+ '现况调查'+ '现况研究')	01/01/1990 - 04/02/2016	Comprehensive search: subject, title, keywords and abstract
**Wanfang**	27/11/2016	Not applicable	Journal articles, Dissertations, Conference articles	(主题:(干眼)+ 主题:(干眼症)+ 主题:(干眼病)) * (主题:(发病率) + 主题:(发生率) + 主题:(患病率) +主题:(罹患率) + 主题:(现患率) + 主题:(死亡率) +主题:(病死率) + 主题:(流行) + 主题:(负担)+ 主题:(现况调查) + 主题:(现况研究))	1990-2016	Comprehensive search: subject (including title, keywords and abstract)
**SinoMed**	27/11/2016	Medicine & Public Health	All journals	((干眼) OR (干眼症) OR (干眼病)) AND ((发病率) OR (发生率) OR (患病率) OR (罹患率) OR (现患率) OR (死亡率) OR (病死率) OR (流行) OR (负担) OR (现况调查) OR (现况研究))	1990-2016	Comprehensive search: title, keywords and abstract
**PubMed**	27/11/2016	Not applicable	Not applicable	((dry eye) AND (China OR Chinese) AND (inciden* OR prevalen* OR morbidity OR mortality OR epidemiology)) AND ("1990/01/01"[Date - Publication] : "2016/11/27"[Date - Publication])	01/01/1990 - 27/11/2016	Comprehensive search: all fields

**Embase**	27/11/2016	Not applicable	Not applicable	1<br>2<br>3<br>4<br>5<br>6<br>7<br>8<br>9<br>10	dry eye.mp. or exp dry eye/<br>Chin*.mp.<br>incidence/ or inciden*.mp.<br>prevalence/ or prevalen*.mp.<br>morbidity/<br>mortality/<br>epidemiology/<br>3 or 4 or 5 or 6 or 7<br>1 and 2 and 8<br>limit 9 to yr="1990 -Current"	01/01/1990 - 27/11/2016	Comprehensive search: all fields
**Medline**	27/11/2016	Not applicable	Not applicable	1<br>2<br>3<br>4<br>5<br>6<br>7<br>8<br>9<br>10	dry eye.mp. or exp Dry Eye Syndromes/<br>Chin*.mp.<br>incidence/ or inciden*.mp.<br>prevalence/ or prevalen*.mp.<br>morbidity/<br>mortality/<br>epidemiology/<br>3 or 4 or 5 or 6 or 7<br>1 and 2 and 8<br>limit 9 to yr="1990 -Current"	01/01/1990 - 27/11/2016	Comprehensive search: all fields

Table S2. Quality score scale for assessing the risk of bias.

Bias type	Low risk (score=2)	Moderate risk (score=1)	High risk (score=0)
Selection (sample population)	1) Sample from general population, not a select group;<br>2) Consecutive unselected population;<br>3) Rationale for case and control selection explained.	1) Sample selected from large population but selection criteria not defined;<br>2) Sample selection ambiguous but may be representative;<br>3) Rationale for cases and controls not explained;<br>4) Eligibility criteria not explained;<br>5) Analysis to adjust for sampling strategy bias.	1) Highly select population making it difficult to generalise finding;<br>2) Sample selection ambiguous and sample unlikely to be representative.
Selection (sample size)	1) Sample size calculation performed and adequate.	1) Sample size calculation performed and reasons for not meeting sample size given;<br>2) Sample size calculation not performed but all eligible persons studied.	1) Sample size estimation unclear or only sub-sample studied.
Selection (participation rate)	1) High response rate (>85%).	1) Moderate response rate (70–85%).	1) Low response rate (<70%);<br>2) Response rate not reported.
Performance bias (outcome assessment)	1) Diagnosis using consistent criteria and direct examination.	1) Assessment from administrative database or register;<br>2) Assessment from hospital record or interviewer.	1) Assessment from non-validated data or generic estimate from the overall population.
Performance bias (analytical methods to control for bias)	1) Analysis appropriate for the type of sample (subgroup analysis/regression etc.).	1) Analysis does not account for common adjustment.	1) Data confusing.

Table S3. Characteristics of the included studies.

Study	Province	Sex	Setting	Study year	Sampling	Assessment	Definition	Age range	Sample size	Cases	DED prevalence by symptoms and signs	DED prevalence by symptoms
Liang ST, et al. (2013)	Hebei	Both	Rural	2012	Cluster random sampling	Symptom, BUT, SIT, fluorescein	(++) BUTS<5s or SIt< 5s; (+) BUTS≤ 10s or SIt≤ 10s or FS≥1; symptoms+one (++) or Symptoms+two (+)	10-14	3007	858	Yes	No
Deng CM, et al. (2015)	Hubei	Both	Urban	2014	Clustered-stratified random sampling	Symptom, BUT, SIT, fluorescein	(++) BUTS≤ 5s or SIt≤ 5s; (+) BUTS≤ 10s or SIt≤ 10s or FS≥1; symptoms+one (++) or Symptoms+two (+)	45-75	420	173	Yes	No
Liu YB, et al. (2015)	Shanxi	Both	Mixed	2013	Random sampling	Symptom, BUT, SIT, fluorescein	(++) BUTS≤ 5s or SIt≤ 5s; (+) BUTS≤ 10s or SIt≤ 10s or FS≥1; symptoms+one (++) or Symptoms+two (+)	9-13	2326	482	Yes	No
Tian J (2013)	Gansu	Both	Mixed	2012	Cluster random sampling	Symptom, BUT, SIT, fluorescein	(++) BUTS<5s or SIt< 5s; (+) BUTS≤ 10s or SIt≤ 10s or FS≥1; symptoms+one (++) or Symptoms+two (+)	6-17	2548	303	Yes	No
Zhuang SJ, et al. (2012)	Guangdong	Both	Urban	2010	Random sampling	Symptom, BUT, SIT, fluorescein	(++) BUTS≤ 5s or SIt≤ 5s; (+) BUTS<10s or SIt< 10s or FS≥1; symptoms+one (++) or Symptoms+two (+)	14-90	2475	154	Yes	No
Xiao XL, et al. (2009)	Guangxi	Both	Urban	2008	Stratified random sampling	Symptom, BUT, SIT, fluorescein	(++) BUTS≤5s or SIt≤ 5s; (+) BUTS≤ 10s or SIt≤ 10s or FS≥1;	<20-60+	10687	1179	Yes	Yes

							symptoms+one (++) or Symptoms+two (+)					
Yang LD, et al. (2014)	Hebei	Both	Mixed	2012	Clustered-stratified random sampling	Symptom, BUT, SIT, fluorescein	(++) BUTS≤5s or SIt≤ 5s; (+) BUTS≤ 10s or SIt≤ 10s or FS≥1; symptoms+one (++) or Symptoms+two (+)	6-14	3150	858	Yes	No
Wei CL, et al. (2013)	Hebei	Both	Urban	2012	Stratified random sampling	Symptom, BUT, SIT, fluorescein	(++) BUTS<5s or SIt< 5s; (+) BUTS≤ 10s or SIt≤ 10s or FS≥1; symptoms+one (++) or Symptoms+two (+)	20-86	870	241	Yes	No
Chu ZD (2011)	Jiangsu	Both	Urban	2010	Cluster random sampling	Symptom, BUT, SIT, fluorescein	(++) BUTS≤5s or SIt≤ 5s; (+) BUTS≤ 10s or SIt≤ 10s or FS≥1; symptoms+one (++) or Symptoms+two (+)	50-95	827	178	Yes	No
Zhou YM (2014)	Shanxi	Both	Rural	2013	Cluster sampling	Symptom, BUT, SIT, fluorescein	(++) BUTS≤5s or SIt≤ 5s; (+) BUTS≤ 10s or SIt≤ 10s or FS≥1; symptoms+one (++) or Symptoms+two (+)	10-86	668	63	Yes	No
Wang J, et al. (2016)	Shandong	Both	Mixed	2013	NS	Symptom, BUT, SIT, fluorescein	(++) BUTS≤5s or SIt≤ 5s; (+) BUTS≤ 10s or SIt≤ 10s or FS≥1; symptoms+one (++) or Symptoms+two (+)	5-72	8145	1803	Yes	No
Zhang XX (2014)	Xinjiang	Both	Urban	2013	Cluster random sampling	Symptom, BUT, SIT, fluorescein	(++) BUTS≤5s or SIt≤ 5s; (+) BUTS< 10s or SIt< 10s or FS≥1; symptoms+one (++) or Symptoms+two (+)	8-69	565	152	Yes	No
Tian YJ, et al. (2009)	Shanghai	Both	Urban	2008	Cluster random sampling	Symptom, BUT, SIT, fluorescein	(++) BUTS<5s or SIt< 5s; (+) BUTS≤ 10s or SIt≤ 10s or FS≥1;	60+	746	185	Yes	No

							symptoms+one (++) or Symptoms+two (+)					
Zou XR, et al. (2016)	Shanghai	Both	Urban	2013	Cluster random sampling	Symptom, BUT, SIT, fluorescein	(++) BUTS≤5s or SIt≤ 5s; (+) BUTS≤ 10s or SIt≤ 10s or FS≥1; symptoms+one (++) or Symptoms+two (+)	60+	2058	295	Yes	No
Tian YJ (2009)	Shanghai	Both	Urban	2008	Random sampling	Symptom, BUT, SIT, fluorescein	(++) BUTS≤5s or SIt≤ 5s; (+) BUTS≤ 10s or SIt≤ 10s or FS≥1 ; symptoms+one (++) or Symptoms+two (+)	20-95	1085	326	Yes	Yes
Lei HY (2012)	Guangxi	Both	Mixed	2009	Cluster random sampling	Symptom, BUT, SIT, fluorescein	(++) BUTS≤ 5s or SIt≤ 5s; (+) BUTS<10s or SIt< 10s or FS>3; symptoms+one (++) or Symptoms+two (+)	Middle and high school students	2358	157	Yes	No
Yang ZL (2006)	Tibet	Both	Mixed	2005	Random sampling	Symptom, BUT, SIT, fluorescein	(++) BUTS≤ 5s or SIt≤ 5s; (+) BUTS<10s or SIt< 10s or FS≥2; symptoms+one (++) or Symptoms+two (+)	40-79	680	131	Yes	No
Gong YY, et al. (2015)	Xinjiang	Both	Urban	2013	Random sampling	Symptom, BUT, SIT, fluorescein	symptoms and (BUTS≤ 10s or SIt≤ 10s or FS≥1 or MGD)	19-93	829	179.893	Yes	No
Ran WJ (2015)	Chongqing	Both	Urban	2013	Health check-up	Symptom, BUT, SIT, fluorescein	symptoms and signs	18-60	16548	1081	Yes	No
Labbe A, et al. (2013)	beijing	Both	Mixed	2006	Random sampling	Symptom, BUT, SIT, fluorescein	BUTS< 10s and/or SIt<5s and symptoms	50-91	1456	241	Yes	No
Zhang Y, et al. (2010)	Shanghai	Both	Urban	2007	Cluster random sampling	Symptom, BUT, SIT, fluorescein	(++) BUTS≤ 5s or SIt≤ 5s; (+) BUTS<10s or SIt< 10s or FS≥3;	45-75	1601	219	Yes	No

							symtoms+one (++) or Symtoms+two (+)					
He HQ, et al. (2007)	Jiangsu	Both	Mixed	2007	NS	Symptom, BUT, SIT, fluorescein	(++) BUTS≤ 5s or SIt≤ 5s; (+) BUTS<10s or SIt< 10s or FS>3; symtoms+one (++) or Symtoms+two (+)	18-90	393	89	Yes	Yes
Wang WX, et al. (2015)	Yunnan	Mixed	Mixed	2013	NS	Symptom, BUT, SIT, fluorescein	(++) BUTS≤ 5s or SIt≤ 5s; (+) BUTS<10s or SIt< 10s or FS>3; symtoms+one (++) or Symtoms+two (+)	19-85	380	86	Yes	No
Liu YB, et al. (2016)	Shanxi	Both	Mixed	2012	NS	Symptom, BUT, SIT, fluorescein	(BUTS≤ 10s or SIt≤ 10s or FS≥1) + Symptoms	42-86	2137	679	Yes	No
Chen F (2016)	Jiangxi	Both	Mixed	2014	Random sampling	Symptom, BUT, SIT, fluorescein, Tear osmolarity	only symptoms	20-69	900	350	No	Yes
Sun YZ, et al. (2010)	Liaoning	Both	Mixed	2007	Random sampling	Symptom, BUT, SIT, fluorescein	only symptoms	20+	1133	336	No	Yes
Han YL et al. (2009)	Qinghai	Both	Rural	2008	Random sampling	Symptom, BUT, SIT, fluorescein	only symptoms	17-73	1500	887	No	Yes
Zhang Y, et al. (2014)	Shandong	Both	Mixed	2010	Cluster random sampling	Symptom, BUT, SIT, fluorescein	previous DES or only symptoms	High school students	1885	447	No	Yes
Zhao JF (2016)	Zhejiang	Mixed	Mixed	2015	Cluster random sampling	Symptom, BUT, SIT, fluorescein	only symptoms	High school students	1458	397	No	Yes
Lu P, et al. (2008)	Qinghai	Both	Mixed	2006	Clustered-stratified random sampling	Symptom, BUT, SIT, fluorescein	only symptoms	40+	1840	965	No	Yes

Jie Y, et al. (2009)	beijing	Both	Mixed	2001	Random sampling	Symptom, BUT, SIT, fluorescein	only symptoms	40-84	1957	411	No	Yes
Lin PY, et al. (2003)	Taiwan	Both	Mixed	2000	Random sampling	Symptom, BUT, SIT, fluorescein	only symptoms reported	65-91	1361	495	No	Yes
Guo B, et al. (2010)	Qinghai	Both	Mixed	2006	Clustered-stratified random sampling	Symptom, BUT, SIT, fluorescein	only symptoms	40+	1816	909	No	Yes
Hua R, et al. (2014)	northeast China	Mixed	Rural	2007	Cluster sampling	Symptom, BUT, SIT, fluorescein	only symptoms	12-88	2262	1710	No	Yes

Note: NS, not specified.

Table S4. Quality scores for assessing the risk of bias in the included studies.

Study	Title	Quality score					
		Sample population	Sample size	Participation	Outcome assessment	Analytical methods	Total scores
Liang ST, et al. (2013)	沧州市学龄儿童干眼的流行病学调查	2	2	2	2	2	10
Deng CM, et al. (2015)	城市中老年人电子产品使用情况与干眼相关因素的分析	2	2	2	2	2	10
Liu YB, et al. (2015)	汾阳市 9～14 岁儿童干眼患病现状及危险因素分析	2	2	2	2	2	10
Tian J (2013)	甘肃省甘南藏族自治州舟曲县藏、汉族小学生干眼症的流行病学调查	2	2	2	2	2	10
Zhuang SJ, et al. (2012)	广东省惠东县社区人群干眼的流行病学调查	2	2	2	2	2	10
Xiao XL, et al. (2009)	广西柳州市普通人群干眼症流行病学的调查	2	1	2	2	2	9
Yang LD, et al. (2014)	河北省沧州城区 6～14 岁学龄儿童眼病现况调查	2	1	2	2	2	9
Wei CL, et al. (2013)	石家庄某社区干眼症患病率调查	2	2	2	2	2	10
Chu ZD (2011)	无锡市干眼症流行病学调查	2	2	2	2	2	10
Zhou YM (2014)	长治市神龙湾村自然人群干眼患病率年龄相关性及 Meta 分析	2	1	2	2	2	9
Wang J, et al. (2016)	2011 /2014 年德州市特定人群干眼症流行趋势及相关危险因素研究	2	1	2	2	2	9
Zhang XX (2014)	克拉玛依市区干眼症患病情况的流行病学调查分析	2	2	1	2	2	9
Tian YJ, et al. (2009)	上海市北新泾社区 60 岁及以上人群干眼的流行病学调查	2	2	2	2	2	10
Zou XR, et al. (2016)	上海市奉贤区奉城镇老年人干眼的流行病学调查	2	1	2	2	2	9

Tian YJ (2009)	上海市江宁街道 20 岁及以上人群干眼的流行病学调查	2	2	2	2	2	10
Lei HY (2012)	广西横县地区青少年干眼症的调查分析	2	1	2	2	2	9
Yang ZL (2006)	西藏地区干眼症发病情况的初步调查与分析	1	1	0	2	2	6
Gong YY, et al. (2015)	新疆喀什地区维吾尔族人群干眼流行病学调查	1	1	0	2	2	6
Ran WJ (2015)	重庆市城市居民眼部健康状况调查分析	1	1	0	2	2	6
Labbe A, et al. (2013)	Dry eye disease, dry eye symptoms and depression: the Beijing Eye Study	1	1	1	2	2	7
Zhang Y, et al. (2010)	上海市花木社区中老年人群干眼的患病率调查分析	2	1	0	2	2	7
He HQ, et al. (2007)	体检人群干眼症患病率初步调查	1	1	0	2	2	6
Wang WX, et al. (2015)	体检人群干眼症患病率的初步调查	1	1	0	2	2	6
Liu YB, et al. (2016)	中老年人群干眼患病率及相关危险因素	1	1	0	2	2	6
Chen F (2016)	景德镇地区干眼症的流行病学调查	2	1	0	2	2	7
Sun YZ, et al. (2010)	辽宁沿海农村与城镇居民干眼症发病情况初探	2	1	0	2	2	7
Han YL et al. (2009)	青海高原牧民干眼症的调查分析	2	1	0	2	2	7
Zhang Y, et al. (2014)	山东省寿光市高中生干眼患病率及影响因素	2	2	2	2	2	10
Zhao JF (2016)	职高生干眼症患病率与影响分析	2	1	2	2	2	9
Lu P, et al. (2008)	Dry Eye Syndrome in Elderly Tibetans at High Altitude A Population-based Study in China	2	2	1	2	2	9
Jie Y, et al. (2009)	Prevalence of dry eye among adult Chinese in the Beijing Eye Study	2	1	0	2	2	7
Lin PY,et al. (2003)	Prevalence of Dry Eye among an Elderly Chinese Population in Taiwan The Shihpai Eye Study	2	1	2	2	2	9

Guo B,et al. (2010)	Prevalence of Dry Eye Disease in Mongolians at High Altitude in China: The Henan Eye Study	2	1	2	2	2	9
Hua R, et al. (2014)	Discrepancy between subjectively reported symptoms and objectively measured clinical findings in dry eye:a population based analysis	2	1	2	2	2	9

Table S5. Age- and sex-specific prevalence of DED by symptoms and signs in the six Chinese regions.

Age range	North China		Northeast China		East China		South Central China		Southwest China		Northwest China		China	
	**Male**	**Female**	**Male**	**Female**	**Male**	**Female**	**Male**	**Female**	**Male**	**Female**	**Male**	**Female**	**Male**	**Female**
5-9 years	10.10 (7.25-13.91)	12.50 (9.08-16.95)	13.66 (8.56-21.09)	16.74 (10.67-25.28)	5.83 (4.43-7.63)	7.29 (5.61-9.43)	4.29 (2.90-6.29)	5.39 (3.68-7.82)	4.84 (3.46-6.74)	6.07 (4.38-8.36)	9.27 (6.86-12.41)	11.49 (8.61-15.18)	6.35 (4.51-8.87)	8.01 (5.76-11.05)
10-14 years	11.06 (7.97-15.14)	13.64 (9.97-18.40)	14.89 (9.40-22.80)	18.20 (11.69-27.22)	6.41 (4.89-8.35)	8.01 (6.18-10.31)	4.72 (3.21-6.90)	5.93 (4.07-8.57)	5.33 (3.82-7.39)	6.68 (4.84-9.15)	10.16 (7.55-13.53)	12.56 (9.46-16.50)	6.98 (4.98-9.72)	8.78 (6.33-12.04)
15-19 years	12.09 (8.76-16.46)	14.88 (10.93-19.94)	16.22 (10.30-24.61)	19.75 (12.78-29.24)	7.04 (5.40-9.14)	8.78 (6.81-11.26)	5.20 (3.54-7.57)	6.52 (4.49-9.38)	5.86 (4.22-8.09)	7.34 (5.33-10.01)	11.12 (8.30-14.73)	13.72 (10.38-17.91)	7.84 (5.61-10.84)	9.77 (7.09-13.32)
20-24 years	13.21 (9.61-17.88)	16.21 (11.97-21.57)	17.64 (11.28-26.52)	21.40 (13.96-31.36)	7.73 (5.95-9.99)	9.63 (7.49-12.29)	5.72 (3.91-8.29)	7.16 (4.95-10.26)	6.45 (4.65-8.86)	8.05 (5.88-10.94)	12.15 (9.12-16.02)	14.96 (11.38-19.42)	8.79 (6.32-12.11)	10.83 (7.89-14.69)
25-29 years	14.41 (10.54-19.39)	17.63 (13.09-23.31)	19.16 (12.34-28.52)	23.15 (15.23-33.56)	8.49 (6.55-10.93)	10.54 (8.24-13.40)	6.29 (4.32-9.09)	7.86 (5.45-11.22)	7.08 (5.13-9.70)	8.83 (6.47-11.95)	13.28 (10.00-17.41)	16.29 (12.45-21.03)	9.70 (7.00-13.30)	11.92 (8.72-16.07)
30-34 years	15.70 (11.54-21.01)	19.14 (14.29-25.15)	20.78 (13.48-30.61)	25.00 (16.59-35.84)	9.30 (7.20-11.94)	11.53 (9.05-14.60)	6.92 (4.76-9.95)	8.63 (6.00-12.25)	7.78 (5.65-10.62)	9.68 (7.12-13.04)	14.48 (10.96-18.90)	17.71 (13.61-22.74)	10.62 (7.68-14.50)	13.02 (9.56-17.46)
35-39 years	17.09 (12.62-22.73)	20.76 (15.58-27.09)	22.49 (14.71-32.80)	26.94 (18.04-38.19)	10.19 (7.92-13.03)	12.61 (9.92-15.89)	7.59 (5.24-10.88)	9.46 (6.60-13.37)	8.53 (6.22-11.61)	10.60 (7.82-14.22)	15.78 (11.99-20.49)	19.24 (14.85-24.55)	11.54 (8.38-15.66)	14.14 (10.43-18.84)
40-44 years	18.57 (13.77-24.55)	22.47 (16.96-29.13)	24.30 (16.03-35.06)	28.98 (19.59-40.60)	11.16 (8.69-14.21)	13.76 (10.87-17.28)	8.34 (5.77-11.90)	10.36 (7.26-14.58)	9.36 (6.84-12.68)	11.60 (8.59-15.49)	17.17 (13.10-22.18)	20.86 (16.17-26.46)	12.65 (9.23-17.07)	15.41 (11.44-20.41)
45-49 years	20.14 (15.02-26.47)	24.28 (18.44-31.26)	26.21 (17.43-37.40)	31.10 (21.23-43.06)	12.20 (9.53-15.49)	15.01 (11.89-18.77)	9.14 (6.34-13.00)	11.34 (7.97-15.88)	10.25 (7.51-13.84)	12.68 (9.41-16.86)	18.66 (14.29-23.98)	22.57 (17.59-28.48)	14.00 (10.24-18.80)	17.02 (12.66-22.42)
	21.82	26.19	28.21	33.31	13.32	16.34	10.02	12.39	11.22	13.84	20.24	24.39	15.61	18.95

50-54 years	(16.35-28.49)	(20.00-33.48)	(18.93-39.80)	(22.96-45.56)	(10.43-16.86)	(12.99-20.36)	(6.97-14.19)	(8.74-17.28)	(8.25-15.09)	(10.31-18.33)	(15.57-25.88)	(19.10-30.59)	(11.45-20.85)	(14.15-24.81)
55-59 years	23.59 (17.77-30.61)	28.19 (21.65-35.79)	30.30 (20.52-42.26)	35.59 (24.79-48.09)	14.53 (11.41-18.34)	17.77 (14.17-22.06)	10.96 (7.65-15.47)	13.54 (9.58-18.78)	12.27 (9.04-16.44)	15.09 (11.28-19.90)	21.92 (16.94-27.88)	26.30 (20.70-32.79)	16.49 (12.18-21.89)	19.99 (15.03-26.00)
60-64 years	25.46 (19.28-32.82)	30.28 (23.40-38.16)	32.48 (22.21-44.76)	37.94 (26.71-50.64)	15.84 (12.46-19.91)	19.30 (15.43-23.87)	11.99 (8.40-16.84)	14.76 (10.49-20.38)	13.40 (9.90-17.88)	16.43 (12.33-21.57)	23.70 (18.39-29.98)	28.31 (22.39-35.08)	17.59 (13.07-23.20)	21.40 (16.19-27.65)
65-69 years	27.43 (20.88-35.12)	32.45 (25.24-40.61)	34.73 (23.99-47.30)	40.35 (28.71-53.18)	17.23 (13.59-21.60)	20.92 (16.78-25.78)	13.10 (9.20-18.32)	16.08 (11.47-22.08)	14.61 (10.83-19.43)	17.87 (13.45-23.34)	25.58 (19.94-32.17)	30.40 (24.17-37.45)	18.87 (14.09-24.73)	22.89 (17.41-29.39)
70-74 years	29.49 (22.58-37.49)	34.71 (27.16-43.10)	37.06 (25.85-49.85)	42.80 (30.80-55.72)	18.72 (14.80-23.39)	22.64 (18.21-27.79)	14.29 (10.07-19.90)	17.49 (12.52-23.89)	15.92 (11.83-21.09)	19.40 (14.65-25.23)	27.55 (21.57-34.45)	32.58 (26.04-39.88)	20.55 (15.41-26.79)	24.67 (18.85-31.48)
75-79 years	31.63 (24.36-39.93)	37.03 (29.17-45.64)	39.44 (27.81-52.41)	45.29 (32.97-58.23)	20.31 (16.10-25.29)	24.46 (19.73-29.91)	15.58 (11.01-21.58)	19.00 (13.66-25.80)	17.32 (12.91-22.84)	21.03 (15.94-27.21)	29.61 (23.30-36.81)	34.84 (28.00-42.38)	22.09 (16.65-28.6)	26.10 (20.08-33.09)
80-84 years	33.86 (26.23-42.43)	39.42 (31.26-48.21)	41.88 (29.86-54.96)	47.81 (35.21-60.70)	21.99 (17.47-27.29)	26.38 (21.34-32.12)	16.95 (12.02-23.37)	20.6 0 (14.87-27.82)	18.82 (14.07-24.71)	22.76 (17.32-29.30)	31.76 (25.11-39.25)	37.17 (30.03-44.92)	23.71 (17.94-30.54)	27.61 (21.36-34.80)
85-89 years	36.16 (28.19-44.97)	41.86 (33.42-50.79)	44.36 (31.98-57.49)	50.33 (37.50-63.12)	23.77 (18.94-29.40)	28.39 (23.04-34.43)	18.43 (13.11-25.26)	22.31 (16.17-29.93)	20.41 (15.30-26.68)	24.58 (18.78-31.48)	33.99 (27.01-41.75)	39.56 (32.14-47.50)	25.45 (19.35-32.58)	29.15 (22.67-36.54)

Table S6. Age-specific prevalence and number of people with DED by symptoms in China in 2010.

**Age range**	**Prevalence of DED by symptoms (%, 95% CI)**	**People with DED by symptoms (million, 95% CI)**
5-9 years	16.36 (11.03-23.58)	11.59 (7.82-16.71)
10-14 years	18.24 (12.42-25.99)	13.66 (9.30-19.47)
15-19 years	20.29 (13.95-28.55)	20.27 (13.93-28.52)
20-24 years	22.51 (15.64-31.28)	28.67 (19.92-39.85)
25-29 years	24.89 (17.48-34.14)	25.14 (17.65-34.49)
30-34 years	27.43 (19.48-37.13)	26.65 (18.92-36.07)
35-39 years	30.13 (21.65-40.24)	35.57 (25.55-47.49)
40-44 years	32.98 (23.98-43.43)	41.14 (29.91-54.18)
45-49 years	35.96 (26.47-46.69)	37.97 (27.95-49.30)
50-54 years	39.04 (29.11-49.98)	30.75 (22.92-39.36)
55-59 years	42.22 (31.89-53.28)	34.33 (25.93-43.33)
60-64 years	45.47 (34.80-56.57)	26.68 (20.42-33.19)
65-69 years	48.75 (37.82-59.80)	20.04 (15.55-24.59)
70-74 years	52.05 (40.93-62.96)	17.16 (13.50-20.76)
75-79 years	55.32 (44.11-66.02)	13.20 (10.52-15.75)
80-84 years	58.55 (47.33-68.96)	7.83 (6.33-9.22)
85-89 years	61.71 (50.56-71.76)	3.48 (2.85-4.04)
Overall (5-89 years)	31.40 (23.02-41.13)	394.13 (288.99-516.30)

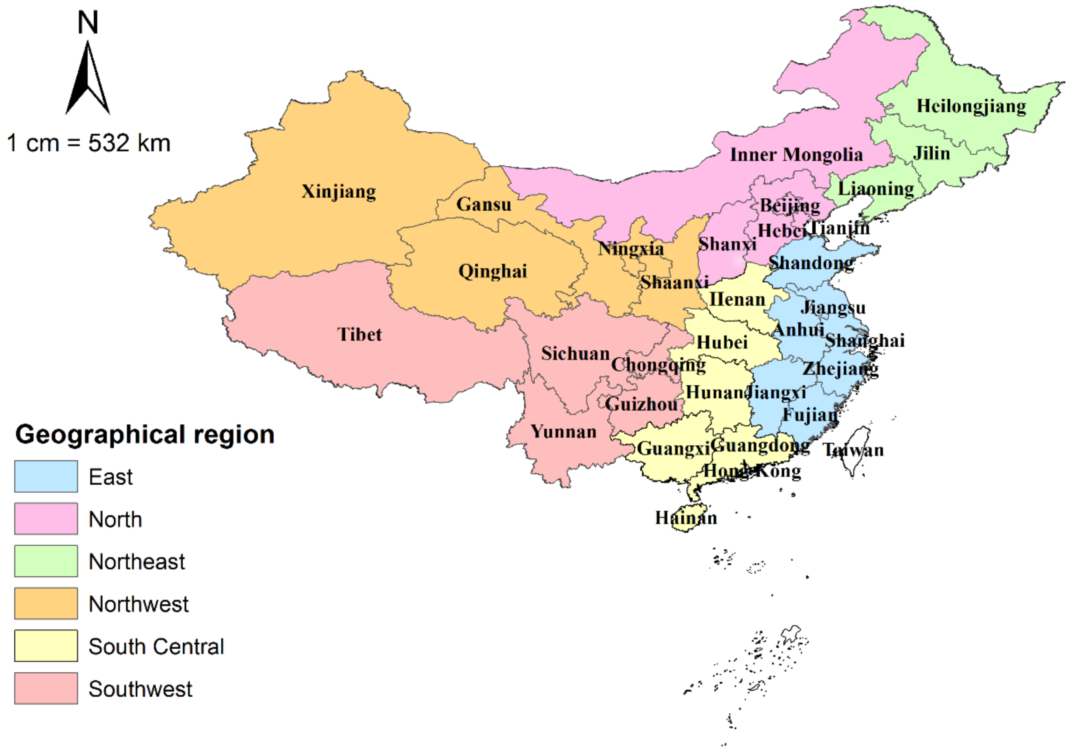

Figure S1. Map of China and the six geographic regions. The map was created using ArcMap version 10.1 (www.esri.com/software/arcgis).

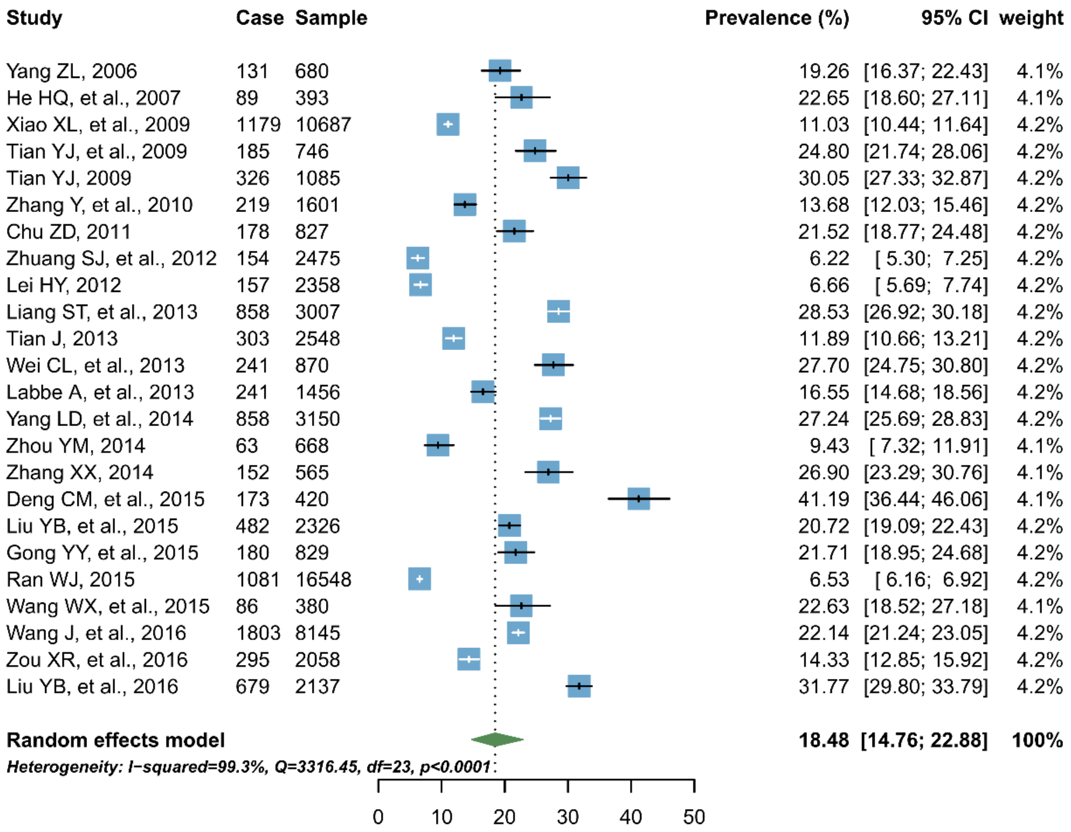

Figure S2. Forest plot of the 24 studies reported prevalence of DED by symptoms and signs.

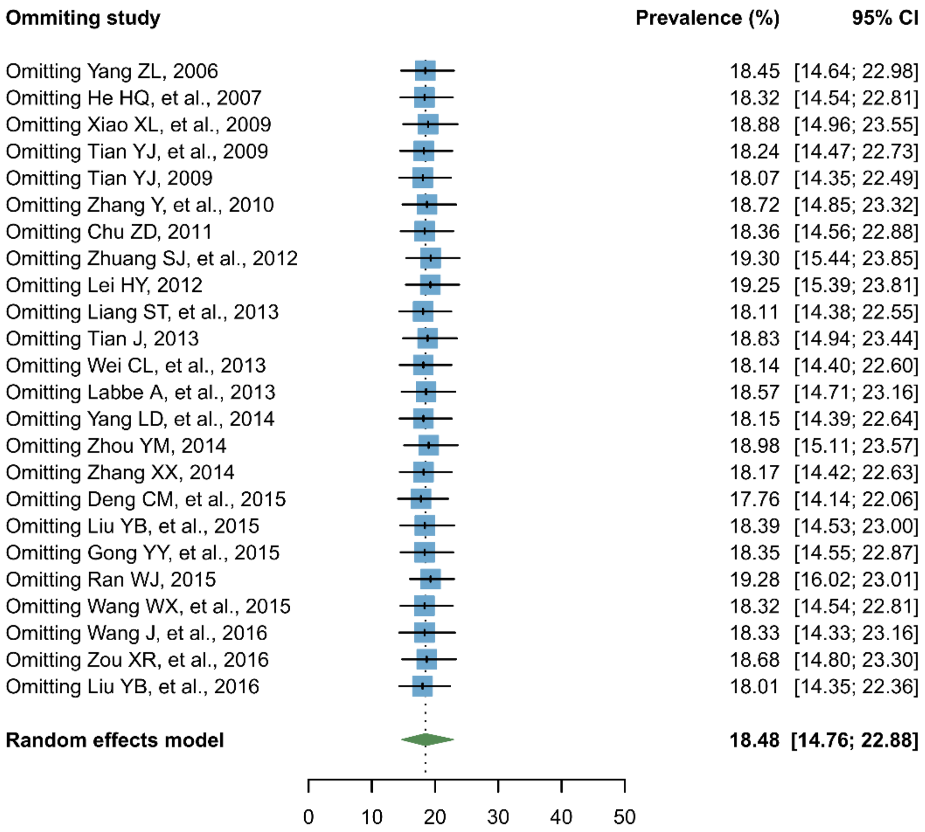

Figure S3. Leave-one-out sensitivity analysis of the influence of single study on the pooled prevalence of DED by symptoms and signs.

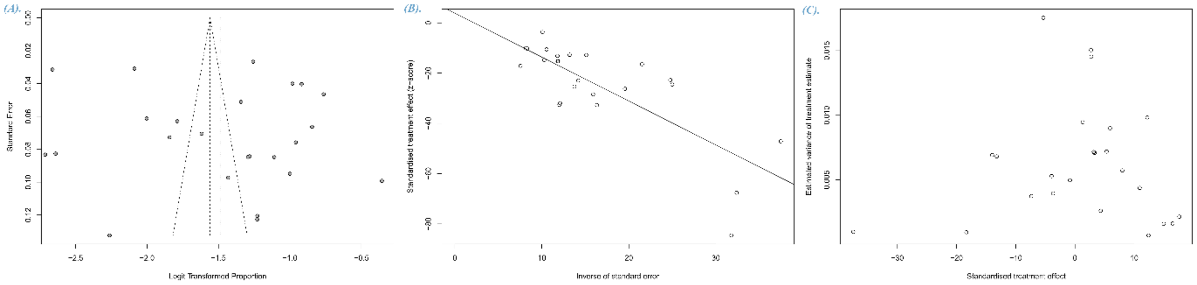

Figure S4. Publication bias of the 24 studies prevalence of DED by symptoms and signs.
Note: (A) Funnel plot; (B) Egger’s test; (C) Begg’s test.

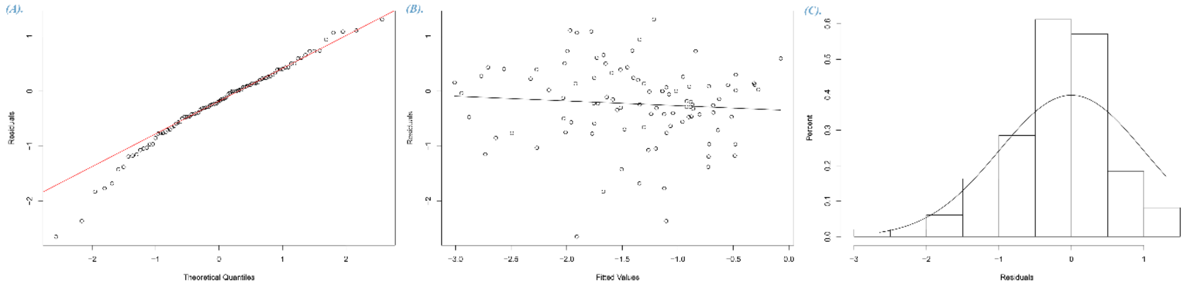

Figure S5. Diagnostic plots of standardized residuals for logit prevalence of DED by symptoms and signs.

Note: (A) QQ plot of residuals; (B) Predicted vs. standardized residuals; (C) Histogram of residuals.

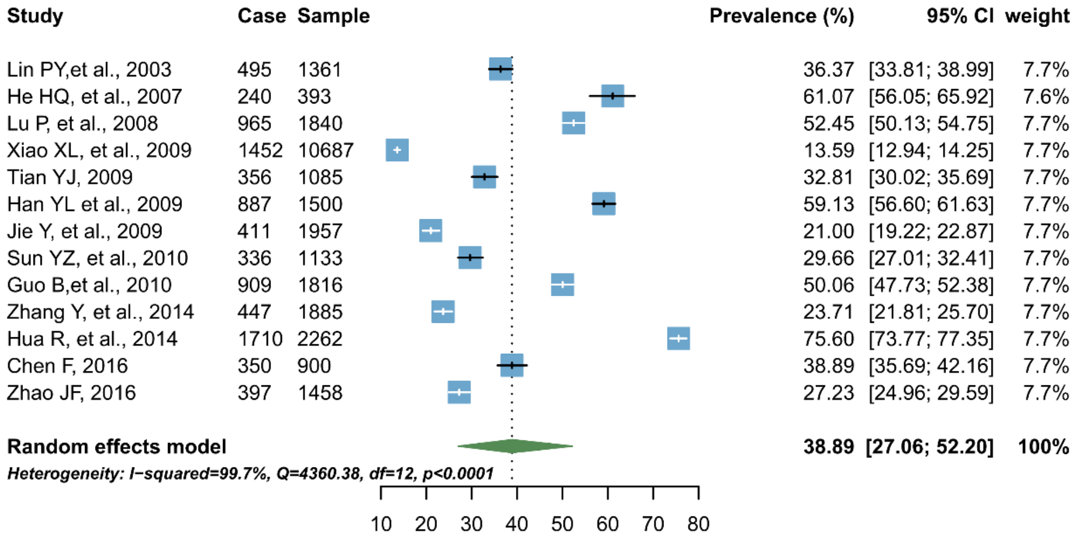

Figure S6. Forest plot of the 13 studies reported prevalence of DED by symptoms.

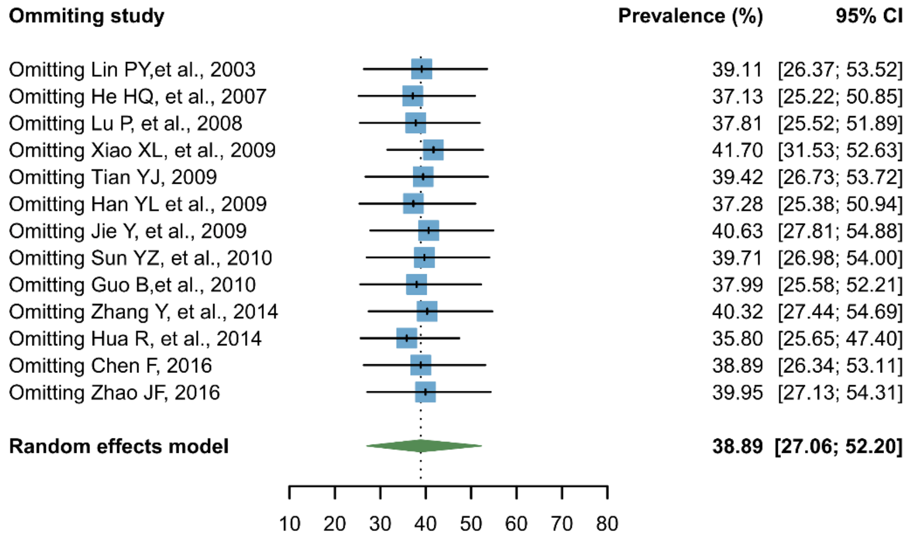

Figure S7. Leave-one-out sensitivity analysis of the influence of single study on the pooled prevalence of DED by symptoms.

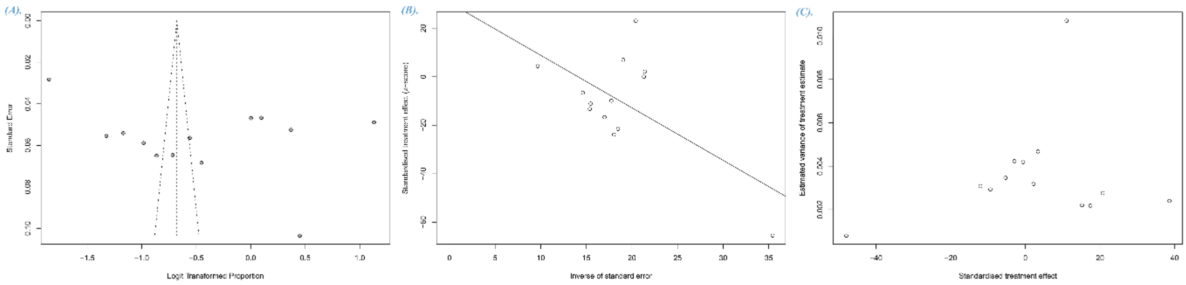

Figure S8. Publication bias of the 24 studies prevalence of DED by symptoms.
Note: (A) Funnel plot; (B) Egger's test; (C) Begg's test.

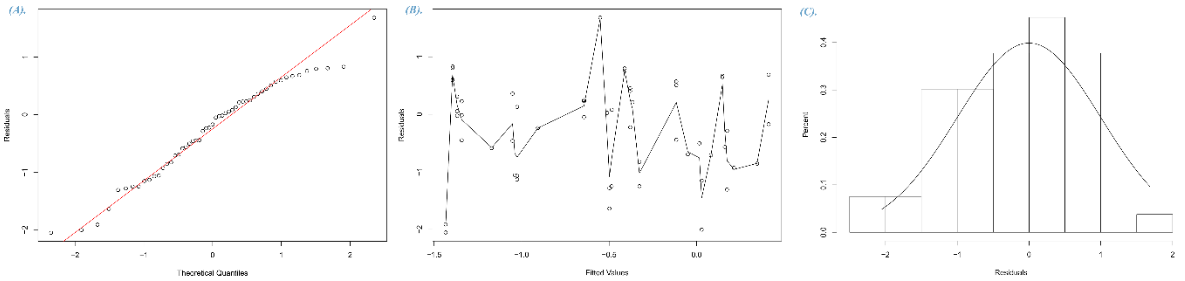

Figure S9. Diagnostic plots of standardized residuals for logit prevalence of DED by symptoms.
Note: (A) QQ plot of residuals; (B) Predicted vs. standardized residuals; (C) Histogram of residuals.
